# U-AttentionFlow: A Multi-Scale Invertible Attention Network for OLTC Anomaly Detection Using Acoustic Signals

**DOI:** 10.3390/s25196244

**Published:** 2025-10-09

**Authors:** Donghyun Kim, Hoseong Hwang, Hochul Kim

**Affiliations:** Department of Medical Artificial Intelligent, Eul-Ji University, Seongnam-si 13135, Gyeonggi-do, Republic of Korea; kdh11191224@g.eulji.ac.kr (D.K.);

**Keywords:** OLTC, anomaly detection, flow-based models, U-Net, one-class classification, attention mechanism, multi-scale feature fusion

## Abstract

The On-Load Tap Changer (OLTC) in power transformers is a critical component responsible for regulating the output voltage, and the early detection of OLTC faults is essential for maintaining power grid stability. In this paper, we propose a one-class deep learning anomaly detection model named “U-AttentionFlow” based on acoustic signals from the OLTC operation. The proposed model is trained exclusively on normal operating data to accurately model normal patterns and identify anomalies when new signals deviate from the learned patterns. To enhance the ability of the model to focus on significant features, we integrate the squeeze-and-excitation (SE) block and Convolutional Block Attention Module (CBAM) into the network architecture. Furthermore, static positional encoding and multihead self-attention (MHSA) are employed to effectively learn the temporal characteristics of time-series acoustic signals. We also adopted a U-Flow-style invertible multiscale coupling structure, which integrates features across multiple scales while ensuring the invertibility of the model. Experimental validation was conducted using acoustic data collected under realistic voltage and load conditions from actual ECOTAP VPD OLTC equipment, resulting in an anomaly detection accuracy of 99.15%. These results demonstrate the outstanding performance and practical applicability of the U-AttentionFlow model for OLTC anomaly detection.

## 1. Introduction

In recent years, artificial intelligence (AI) has advanced rapidly, resulting in significant transformations across various industrial sectors, including the electric power industry. AI has notably improved efficiency in areas such as data analysis, predictive maintenance, and automated operational control within power systems [1]. However, as the implementation of AI technologies has expanded, electricity consumption has increased substantially, posing new challenges in maintaining power grid stability. For instance, AI-driven high-performance computing and data center operations lead to considerable electricity consumption, imposing substantial burden on existing power infrastructure [2].

Despite the rapid increase in electricity demand, significantly expanding the power generation capacity is not a realistic solution owing to numerous constraints. Increasing the proportion of renewable energy sources requires extensive time and substantial investment. Therefore, there is an urgent need to develop technologies that can efficiently utilize the existing power infrastructure, including smart grid technologies, power transformer management, and demand-side management (DSM) [3,4]. In particular, stable operation of power transformers is essential for maintaining power grid stability.

Power transformers are critical components of power grids that enable efficient transmission of electricity by converting voltage levels. The reliable operation of the transformers directly affects the overall grid reliability. Among the transformer components, the On-Load Tap Changer (OLTC) plays a crucial role in adjusting the transformer output voltage to minimize voltage fluctuations resulting from load variations. An OLTC is essential for maintaining power system stability because it responds to load changes by regulating the transformer taps in real time [5,6]. However, owing to its inherent operating characteristics, the OLTC frequently experiences high load currents, increasing the likelihood of switching contact deterioration and potential faults.

The OLTC uses switching contacts through which high currents flow during the tap-changing process to adjust the output voltage of the transformer. Owing to repeated operation, these switching contacts gradually wear out, resulting in reduced conductivity. Damage to the contacts can eventually lead to OLTC failure, significantly affecting the overall reliability of the transformer [7,8]. According to various studies, OLTC failures are identified as one of the primary causes of transformer failures, with approximately 30–40% of all transformer faults attributed to OLTC-related issues.

Therefore, regular condition monitoring and fault diagnosis are essential for reliable operation of OLTCs. Currently, various data types are employed to diagnose OLTC faults, including dissolved gas analysis (DGA), temperature, vibration, voltage, current, and acoustic signals [9]. DGA involves analyzing the gases generated in the insulating oil to detect contact wear or arc occurrence within the OLTC [10]. Temperature data are crucial for diagnosing overheating issues caused by contact degradation or excessive loads [11]. Vibration data can effectively identify mechanical issues or contact wear occurring during the OLTC switching operations [12,13]. Voltage data were used to verify whether the OLTC accurately regulated the transformer output voltage. Current data enable the detection of anomalies by analyzing load variations and current flows during the OLTC operation. Finally, acoustic data facilitate the early detection of contact arcs and mechanical abnormalities by analyzing the noise patterns generated during OLTC operations [14,15].

Dissolved Gas Analysis (DGA) enables the indirect assessment of insulation conditions within OLTCs and the identification of arcs or overheating at the contacts by analyzing the concentrations of various gases produced inside the transformer [10]. This approach is advantageous for preventive maintenance because it allows early detection of incipient faults. Additionally, the specific type of gas detected can indicate the cause and location of the fault [9]. However, a drawback of this method is the delay caused by the time required for gases to accumulate, which limits their effectiveness in responding swiftly to faults. Furthermore, specialized equipment and expertise are necessary for a precise analysis.

Utilizing temperature data allows the real-time monitoring of the thermal conditions within the OLTC. An increase in temperature can serve as a direct indicator of contact wear or overload conditions, thereby facilitating the rapid identification of potential faults [11]. This enables a prompt response and is useful for the long-term tracking of contact conditions. However, one limitation is that temperature variations may indicate faults that are already in progress rather than early-stage detection. Additionally, temperature anomalies do not necessarily signify faults, highlighting the need for a combined analysis with other data types to accurately diagnose OLTC conditions.

Vibration data are highly effective in monitoring the mechanical conditions of OLTCs. Vibration sensors can identify mechanical wear or imbalance issues by detecting and analyzing the vibration patterns generated during OLTC operation [12,13]. These data are particularly valuable in identifying the root causes of mechanical faults. However, a significant limitation is the sensitivity of the vibration data to noise, as various external environmental vibrations may interfere with and negatively influence the diagnostic accuracy. Additionally, vibration data primarily capture mechanical anomalies, offering a limited capability for detecting electrical faults. Furthermore, highly sensitive sensors and complex data processing techniques are required for collecting and analyzing vibration data effectively, which can lead to increased system complexity and higher maintenance costs.

Current data can be effectively utilized to monitor the electrical conditions of the OLTCs. Current sensors continuously measure the load current flowing through the OLTC, thereby enabling the detection of abnormal current variations. This approach is particularly effective for diagnosing electrical issues such as short circuits or overload conditions. One of the main advantages of using current data is its real-time collection capability, which allows for immediate fault diagnosis. However, current data are sensitive to specific types of electrical faults; therefore, they have limitations in detecting mechanical or acoustic anomalies. Moreover, distinguishing the exact causes of current variations can be challenging, requiring additional data or complex analytical processes for an accurate diagnosis. Furthermore, current sensors are susceptible to electromagnetic interference (EMI) and noise, necessitating supplementary processing techniques, such as noise filtering, to ensure data accuracy.

Voltage data can be effectively utilized to monitor the output voltage of an OLTC and predict potential faults. These data are useful for verifying whether the OLTC is operating normally and maintaining the output voltage within specified limits. As voltage measurements reflect the overall electrical condition of the transformers, they are essential for assessing the electrical stability. However, voltage data may not directly detect other types of faults such as contact wear or abnormal increases in load current. It predominantly identifies electrical fluctuations and has limited capability in diagnosing nonelectrical issues, such as mechanical wear or internal defects. Furthermore, voltage fluctuations can be caused by multiple factors, making it difficult to identify a given fault using voltage data alone. Additionally, voltage data may be influenced by disturbances occurring elsewhere in the power grid, potentially complicating the accurate diagnosis of OLTC issues [14].

Dynamic resistance measurement (DRM) is a complementary offline diagnostic technique often used to assess OLTC condition. In a DRM test, a small direct current is injected through the tap changer and the resulting current and/or voltage is recorded as a function of time during the switching operation. Analyzing the ripple in the current–time curve and deviations in the calculated resistance–time plot allows detection of make-before-break problems, changes in contact timing, and variations in transition resistor values that static resistance tests cannot reveal. However, DRM must be performed with the transformer deenergized, and interpretation of the results can be challenging because the inductance of the transformer windings and the magnitude of the test current influence the current waveform. Dynamic current curves often need to be compared with reference signatures, and when measurements are made with the secondary winding open, large winding inductance causes slow current variation that can obscure subtle mechanical anomalies such as contact bounce or friction [9]. The inherent limitations of each data type imply that no single type of data is sufficient for a comprehensive fault diagnosis. Unlike dissolved gas analysis, temperature, vibration, current, and voltage data, acoustic data uniquely offer the potential for complete and reliable fault diagnosis of OLTCs [15].

Acoustic data have emerged as critical tools for comprehensively assessing the conditions of OLTCs [16]. By analyzing various acoustic signals generated within OLTCs, this approach enables the early detection of fault indications and facilitates the diagnosis of diverse issues such as switching contact wear, electrical arcs, and mechanical friction [17,18]. A key advantage of acoustic data is their ability to monitor the status of switching contacts in real time, providing exceptionally early detection of faults. Furthermore, acoustic data are comparatively less sensitive to noise than other sensors, and although various external environmental sounds exist, advanced acoustic signal processing techniques can effectively filter out these interferences [19]. Additionally, acoustic data collection is inherently non-contact, unlike vibration or current sensors, significantly reducing risks during installation and maintenance. This non-contact characteristic also leads to reduced infrastructure requirements and associated maintenance costs [19]. Another substantial benefit of acoustic data is their compatibility with older OLTC models, which often have limitations in accommodating new technological installations; acoustic sensors can easily be deployed without such constraints. Moreover, by continuously monitoring abnormal operating sounds, acoustic data can provide proactive warnings before the faults fully manifest, thereby enabling effective preventive maintenance [20].

In conclusion, while dissolved gas analysis and temperature, vibration, current, and voltage data each offer distinct advantages that are useful for specific fault scenarios, acoustic data uniquely provides a comprehensive fault diagnosis without the inherent limitations of these other methods [16,17]. Acoustic data represent a powerful tool capable of thoroughly assessing the condition of the OLTC equipment. Thus, utilizing acoustic data is essential for fault diagnosis and preventive maintenance of OLTCs, significantly enhancing transformer reliability and overall power grid stability.

When utilizing acoustic data for fault diagnosis, three main approaches can be employed: analyzing the data in its original time-series format, transforming it into a spectrogram, or converting it into a scalogram. The direct use of acoustic data as time-series signals is straightforward and requires minimal preprocessing, thus offering advantages for real-time processing. This approach preserves the original characteristics of the signal and facilitates direct signal analysis. However, it is limited to a frequency-domain analysis, which makes it difficult to recognize complex fault patterns, especially in cases involving nonlinear patterns or noisy signals [18].

The spectrogram approach simultaneously analyzes time and frequency information, clearly capturing the frequency variation patterns within the acoustic signals [17]. In addition, spectrograms visually represent signal patterns, simplifying the identification, exploration, and analysis processes. Nonetheless, this method can suffer from information loss during the transformation process, and its fixed time-frequency resolution may limit the accuracy of the analysis in specific frequency bands. Furthermore, the generation of spectrograms incurs additional computational costs.

The scalogram approach, based on wavelet transforms, provides a multi-resolution analysis of time-frequency information. It offers variable time resolution depending on the frequency. High-frequency components are analyzed with precise temporal resolution, whereas low-frequency components are examined over longer durations [20,21]. This capability is particularly beneficial for capturing nonlinear or sudden fault-indicating signal variations. In addition, scalograms can clearly illustrate anomalies occurring across different frequency bands, enabling more accurate and detailed fault pattern recognition. Thus, a scalogram-based analysis is highly effective for capturing complex fault signatures and offers superior diagnostic accuracy owing to its flexible frequency-domain analytical capabilities [21].

Traditional signal processing methods typically focus on frequency analysis and filtering techniques to detect specific patterns. However, they often struggle to effectively capture novel fault patterns or nonlinear changes. These conventional methods generally fail to provide sufficient responsiveness for the early detection of unexpected or infrequently occurring abnormal conditions. By contrast, recent advances in machine learning and deep learning methodologies have significantly improved fault diagnosis capabilities through data-driven pattern recognition [22]. However, these techniques typically require datasets containing both normal and faulty data. In particular, deep learning approaches require substantial amounts of fault data to effectively learn fault patterns, posing a considerable challenge for systems in which faults occur infrequently and data collection is inherently difficult [23].

Securing sufficient fault data is particularly challenging for equipment such as OLTCs, which experience faults at very low frequencies. OLTCs are critical components of power systems, and faults within them can significantly affect the entire system. The low incidence of OLTC failures makes the acquisition of sufficient faulty data difficult, thereby limiting the practical applicability of traditional machine- and deep-learning models [23]. Consequently, there is a pressing need for alternative methodologies capable of diagnosing faults even under limited data conditions.

To address these challenges, one-class learning methods that utilize only normal (healthy) data have attracted increasing attention [24]. one-class learning is designed to detect abnormal conditions based solely on data representing normal operational states, thereby enabling effective fault diagnosis in situations where fault data are scarce. This approach operates by learning normal operational patterns and subsequently identifying anomalies that deviate from these learned patterns, thereby facilitating rapid detection even with limited abnormal data [24,25]. Therefore, one-class learning is a highly suitable solution for systems with low fault occurrence rates.

For OLTC systems, in particular, one-class learning methods can serve as invaluable diagnostic tools. OLTCs are essential for ensuring stable transformer operation, and faults within them may lead to significant economic losses. Given their infrequent failure rates, it is difficult to collect adequate fault data. one-class learning methodologies offer practical advantages by detecting abnormal conditions based solely on normal operational data. This approach can not only predict potential faults but also serve as a proactive diagnostic tool for preventing faults from developing. Furthermore, as a form of unsupervised learning, one-class learning avoids the need for extensive labeling tasks, making it highly applicable in real-world scenarios [25]. Although conventional machine learning methods that rely heavily on labeled datasets consume considerable resources, one-class learning reduces this burden while delivering effective results.

The benefits of one-class learning extend beyond OLTC equipment to various industrial sectors. In particular, in applications involving significant risks, such as semiconductor manufacturing, aircraft engines, and power plant turbines, this methodology is beneficial for monitoring equipment conditions and performing preventive maintenance. In conclusion, one-class learning represents a robust solution for overcoming the limitations of fault diagnosis in environments where fault data are limited. It provides effective fault detection and prediction capabilities in systems characterized by low fault frequency, yet has significant impact when faults occur, enhancing diagnostic reliability and proactively improving overall system stability.

One-class learning can be broadly categorized into Normalizing Flow and memory-efficient embedding methods. Normalizing Flow methods are specialized techniques that transform complex data distributions into simpler distributions [26]. Unlike traditional dimensionality reduction approaches, which merely map high-dimensional data onto lower-dimensional spaces, Normalizing Flow utilizes invertible functions to explicitly reshape the data distribution into a simpler probability distribution, typically a Gaussian distribution. This process clearly models complex data distributions in high-dimensional spaces, making it straightforward to detect abnormal data points as deviations from the normal distribution. Because Normalizing Flow ensures that there is no loss of data/information and maintains invertibility, it effectively preserves data complexity while efficiently detecting anomalies. This capability is particularly advantageous for fault diagnosis applications, where the accurate modeling of normal data distributions enables the effective identification of abnormal data points. Moreover, the probabilistic approach to flow models allows clear boundary definitions for anomaly detection, making Normalizing Flows a powerful tool for one-class learning problems [27].

Memory-efficient embedding methods are designed to enhance the memory efficiency of one-class learning, making them particularly useful when dealing with large-scale datasets [28,29]. These methods aim to reduce memory consumption by embedding data into lower-dimensional spaces, while simultaneously preserving essential data patterns. Unlike conventional dimensionality reduction techniques, memory-efficient embedding not only reduces dimensionality but also learns effective feature spaces specifically tailored for anomaly detection. Particularly in resource-constrained environments that require the processing of large datasets, these methods efficiently differentiate between normal and abnormal data by capturing crucial distinctions within a compact embedding space [28]. Consequently, memory-efficient embedding is highly beneficial for fault diagnosis tasks involving extensive sensor data and clearly separating normal states from abnormal conditions, even under strict memory constraints [29].

Therefore, integrating the strengths of Normalizing Flow and Memory-efficient Embedding methods is expected to yield a superior performance in one-class learning-based fault diagnosis. Recent advances also integrate multiscale attention and residual denoising to handle overlapping and noisy acoustic signals. Zhong et al. introduced a residual denoising and multiscale attention-based weighted domain adaptation network (RDMA-WDAN), which employs residual connections and channel–spatial attention to emphasize fault features in noisy conditions. Such techniques inspire the use of attention mechanisms in our work to address the overlapping and noisy nature of OLTC acoustic signals [30].

To enhance the performance of these networks further, transformer architecture, originally developed for natural language processing, has recently emerged as a prominent approach to overcome the limitations of traditional recurrent neural networks (RNNs) in effectively capturing long-range dependencies among words in sentences. Key components of transformer architectures, namely static positional encoding and multihead self-attention (MHSA), have also been innovatively adopted in computer vision. In particular, the Vision Transformer (ViT) model explicitly encodes spatial structures by dividing images into fixed-size patches, treating these patches as tokens, and adding positional information through fixed embedding [31]. This approach enables parallel and global contextual learning across an entire image, overcoming the constraints imposed by the local receptive fields of convolutional neural netwo32rk (CNN) structures. With the introduction of the MHSA in computer vision, the accuracy of image classification and object recognition tasks has become equal to or even surpassed that of traditional CNN-based models. Initial studies on ViT demonstrated that CNN layers are not indispensable. Notably, MHSA-based models showed superior performance and generalization capabilities on large-scale datasets, such as ImageNet [32].

However, directly applying self-attention to images poses significant computational challenges because their complexity scales quadratically with the input size, thereby presenting efficiency concerns. To address this, models such as the Swin Transformer have been proposed, which employ hierarchical multiscale structures that perform self-attention within localized windows [33]. This approach enables efficient learning of both the global context and local features simultaneously, significantly reducing computational costs and memory requirements while maintaining high accuracy [33]. Consequently, the successful adaptation of Static Positional Encoding and MHSA from natural language processing to computer vision has substantially improved the effective learning of contextual information and detailed image patterns, and transformer architectures have become a new paradigm, overcoming previous limitations in representing long-range relationships. They have significantly enhanced their performance in various computer vision tasks, including object detection, image classification, and anomaly detection [34,35].

Concurrently, U-Net, originally developed as an encoder–decoder structured deep learning model for biomedical image segmentation tasks, has shown exceptional performance in combining global contextual information at low resolution with fine-grained spatial details at high resolution. This model is particularly effective in preserving high-resolution details through symmetrical skip connections between the encoder and decoder paths. Recently, the U-Flow model has adapted the structural characteristics of U-Net for anomaly detection within a Normalizing Flow framework [31,36]. U-Flow introduces a novel invertible multiscale coupling approach that progressively downsamples the image features into coarse and fine resolutions, processes these scales through coupling layers, and subsequently recombines them via invertible upsampling operations. Throughout this process, U-Flow preserves the feature information by employing an invertible skip connection architecture analogous to the skip connections in U-Net, thus ensuring the complete invertibility of transformations [31].

This structure allows U-Flow to model normal image patterns across multiple scales simultaneously, effectively integrating global contextual features and detailed local information. Consequently, U-Flow exhibits significant strengths in detecting both subtle local anomalies and larger structural deviations by effectively combining multiscale features [31]. Owing to the efficient internal flow of information, this model ensures stable training and inference processes and achieves computational efficiency, making it particularly suitable for real-world applications. Furthermore, U-Flow enables precise pixel-level anomaly detection and accurate localization by maintaining feature independence at each scale within its invertible architecture [31]. Drawing inspiration from U-Net and extending its concept, the U-Flow structure has become an effective and practical anomaly detection solution that significantly improves accuracy and computational efficiency by leveraging multiscale information [31,36].

In addition, attention mechanisms continue to be employed to further enhance model performance. The Squeeze-and-Excitation (SE) block is an attention mechanism explicitly designed to model interchannel dependencies, thereby strengthening the feature representations [37]. The SE block compresses global information from each channel through global average pooling and then recalibrates the channel importance by applying nonlinear transformations via two fully connected layers followed by sigmoid activation. These recalibrated channel-wise weights are then multiplied by the original feature maps, emphasizing the important channels while suppressing the less critical ones [37]. Because of its lightweight architecture, the SE block can be easily integrated into various CNN architectures and has demonstrated performance improvements across diverse computer vision tasks, including image classification, object detection, and anomaly detection [37].

The Convolutional Block Attention Module (CBAM) is a lightweight attention module that sequentially applies channel and spatial attention to refine feature maps [38]. The channel attention module first calculates the channel-wise importance using global average pooling and global max pooling, generates channel weights accordingly, and applies them to feature maps. Subsequently, the spatial attention module computes the spatial importance by performing average pooling and max pooling along the channel axis, producing spatial attention maps that further refine the feature maps [38]. Through these dual-stage attention mechanisms, CBAM enables models to effectively learn “what” to focus on and “where”. CBAM can also be conveniently integrated into various CNN architectures, consistently yielding performance enhancements in image classification, object detection, and anomaly detection [38].

In this study, various advanced attention mechanisms are introduced to enhance the existing CS-flow-based anomaly detection model. First, we integrate the SE block and CBAM to clearly capture both channel-wise and spatial importance, significantly strengthening the feature representation. Additionally, static positional encoding and MHSA mechanisms are employed to efficiently learn long-range dependencies and the global context among image features, while preserving spatial structural information. Furthermore, inspired by the U-Net architecture, an invertible multiscale coupling structure, U-Flow, is adopted to seamlessly integrate multiresolution features without loss of information. These structural enhancements greatly improve the detection capability for subtle anomalies and enhance spatial coherence.

Another major contribution of this study is the direct validation of the performance of the proposed model using real-world acoustic data collected from an OLTC equipment under industrial conditions. Through extensive experimentation, the proposed approach demonstrated practical feasibility and superior anomaly detection and localization performance in OLTC devices.

In summary, the key contributions of this study are as follows:

- Enhancement of the feature representation capability of the CS-Flow model by integrating attention-based feature recalibration modules (SE and CBAM) with Transformer-based Static Positional Encoding and Multi-Head Self-Attention.
- Effective preservation of multi-scale information through the introduction of an invertible multi-scale coupling structure inspired by U-Flow.
- The effectiveness and practical applicability of the model were successfully validated through experiments conducted using OLTC acoustic data collected from operational industrial environments.

The remainder of this manuscript is organized as follows. Section 2 discusses the related literature and background specifically on one-class learning methodologies for anomaly detection, highlighting their applicability and challenges. Section 3 provides detailed descriptions of the acoustic dataset collected from OLTC, emphasizing the characteristics and distinctions between normal and abnormal conditions. Section 4 introduces the proposed U-AttentionFlow model architecture, detailing the integration of attention mechanisms, static positional encoding, multi-head self-attention, and invertible multi-scale coupling structures. Section 5 explains the experimental setup, validation methods, and presents comparative evaluations between the proposed method and other existing techniques. Finally, Section 6 summarizes the conclusions drawn from this research and discusses potential directions for future work.

## 2. Related Works

Unsupervised anomaly detection addresses a challenging scenario in which the training images consist exclusively of normal (anomaly free) samples. In such demanding settings, conventional supervised learning algorithms cannot be applied effectively owing to the availability of training data from a single class (normal data). This is often referred to as one-class learning. Methods developed to overcome this challenge differ significantly in the way they recognize distinctions between normal and anomalous data, and are broadly categorized into memory-efficient embedding approaches and Normalizing Flow-based methods.

### 2.1. Memory-Efficient Embedding

Memory-efficient embedding-based anomaly detection methods utilize rich feature representations from pretrained CNN models to learn normal patterns and identify anomalies based on the distance between these learned representations and the features extracted from test images. Prominent approaches in this category include ReConPatch [39], PatchCore [40], and PaDiM [41], each of which models or approximates the normal distribution in the embedding spaces using distinct methodologies.

ReConPatch enhances feature embeddings specifically suited for industrial images by employing contrastive learning. It attaches a linear transformation module to the pretrained CNN patch features, enabling the model to learn embeddings tailored to detect subtle defects. Consequently, it achieves a refined anomaly detection performance through specialized feature representations [39].

PatchCore stores patch-level features extracted from normal images in a memory bank and reduces memory usage by applying coreset subsampling, thus retaining only representative embeddings. During inference, anomalies are identified by calculating the nearest-neighbor distances between the test image patch embeddings and stored embeddings. This approach facilitates rapid anomaly detection through an efficient nearest-neighbor search [40].

PaDiM constructs a multivariate Gaussian distribution for each spatial location using patch embeddings extracted from multiple CNN layers, thereby explicitly modeling the normal patch distribution. It determines anomalies by measuring the Mahalanobis distance at each spatial location to quantify the deviation of the test patches from the modeled distribution. PaDiM simplifies the complexity compared with k-NN-based methods, delivering accurate anomaly detection with enhanced computational efficiency [41].

These embedding-based methods effectively leverage pretrained model knowledge, significantly reduce memory and computational resource requirements, and demonstrate high detection performance, thereby emerging as mainstream techniques in industrial anomaly detection applications [39,40,41].

### 2.2. Normalizing Flow

Normalizing Flow-based anomaly detection methods learn the complex distributions of normal data using Normalizing Flow (NF) models and transform these distributions into simpler probability densities to identify anomalies. NF employs a sequence of invertible transformations, maps arbitrary data distributions to a standard normal distribution, and enables the direct computation of anomaly scores through learned probability density functions. Early studies, such as DifferNet [42], which applies NF at the image level, and CFLOW-AD [43], which extends NF to pixel-level anomaly detection, demonstrated the effectiveness of this approach, prompting the development of more sophisticated variants.

For example, FastFlow leverages a two-dimensional spatial NF to rapidly align single-scale feature maps with a Gaussian distribution, enabling real-time anomaly detection [44]. MSFlow addresses anomalies of varying sizes by processing multiple feature scales through parallel NF pathways, and subsequently merging their outputs. This multiscale strategy allows MSFlow to handle anomalies ranging from small defects to large structural irregularities effectively [45]. Additionally, CSFlow introduces cross-scale connections by integrating multiresolution feature maps within a unified NF model to comprehensively capture both the global context and local details. This approach significantly improves the precision of normal pattern density estimation and enhances sensitivity to subtle anomalies [46]. Similarly, U-Flow integrates multi-scale Transformer features with a U-shaped invertible NF architecture, resulting in a whitened Gaussian latent space that guarantees independence across and within scales, thereby enabling a contrario-based unsupervised threshold selection and achieving state-of-the-art performance across diverse benchmarks [36].

These Normalizing Flow-based methods have recently achieved state-of-the-art performance on benchmarks such as MVTec AD, highlighting their potential as powerful tools for anomaly detection through sophisticated probabilistic modeling of complex normal data distributions [42,43,44,45,46].

## 3. Materials

**Figure 1:**
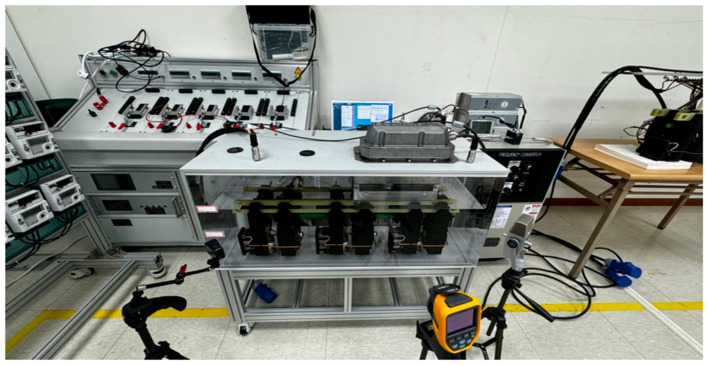
OLTC Experimental Environment.

The experiments conducted in this study utilized ECOTAP VPD^®^ equipment to realistically replicate the operating environment of an OLTC within a laboratory setting in Figure 1. A three-phase configuration was implemented to closely mimic the operating conditions of a transformer by applying a load current of 12 A to each phase. The voltage range was set between 90 V and 120 V, with a target OLTC voltage of 110 V. The ECOTAP device was configured to automatically execute tap changes when deviations from the target voltage were ±5% or more.

**Figure 2:**
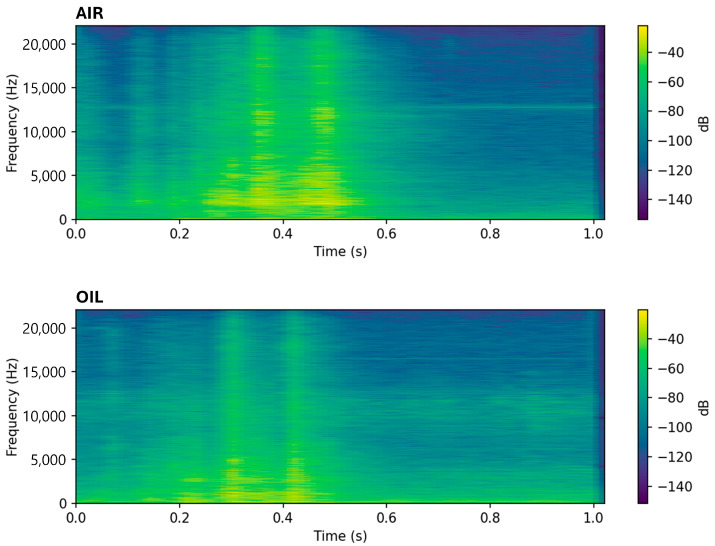
Comparison of DWT Scalograms of OLTC Switching Signals in Air (**top**) and Oil (**bottom**).

In this experiment, air was used as the insulating medium instead of oil, adhering to the warranty conditions of the manufacturer (MR). This decision was aimed at accelerating the OLTC wear through frequent tap switching under oil-free conditions, effectively simulating the accelerated aging scenarios that could arise in actual operational environments. While our warranty-constrained choice of air as the insulating medium allows accelerated wear testing, we acknowledge that acoustic wave propagation in oil-immersed transformers differs from that in air because oil and the steel tank have higher acoustic impedance, faster sound speed and greater attenuation. To verify whether the proposed diagnosis remains valid, additional vibration data were collected in both air and transformer oil. Comparative analysis showed that, despite a lower initial spectral centroid and high-frequency energy ratio in oil, fault evolution produces similar relative patterns: the spectral centroid and high-frequency energy decrease sharply, while low-frequency DWT band energies rise and wavelet entropy falls. This is illustrated in Figure 2, which presents DWT scalograms for representative 1 s segments recorded in air (top) and oil (bottom). The scalograms reveal that both media share the same temporal event structure, but the oil case exhibits markedly stronger low-frequency energy and diminished high-frequency components, consistent with our analysis. These relative trends enable fault detection in oil even though absolute thresholds shift; thus, our method remains applicable when baseline levels are recalibrated for the oil medium.

The acoustic signals generated during OLTC operations were collected using a Brüel & Kjær Type 4955 microphone, a commonly utilized high-sensitivity acoustic sensor. This allowed for the precise capture of detailed acoustic data generated during the tap-changing process. The Brüel & Kjær Type 4955 microphone used in this study has a frequency response of 10 Hz to 20 kHz and an overall noise floor of 6.5 dBA. To capture the full range of frequency content in the OLTC acoustic signals, waveforms were recorded at a 44.1 kHz sampling rate, giving 44,100 samples per 1 s segment and preserving the high-frequency components of the signal.

Each one-second recording contained exactly two tap-change operations: one make and one break cycle triggered when the ECOTAP device detected a ±5% deviation from the target voltage. We chose not to extract shorter segments corresponding to individual switching cycles because the 1 s duration encompasses the complete make–break sequence and preserves the temporal context needed for our analysis. Preliminary tests with sub-second segments did not improve classification performance and, in some cases, degraded it, as the model lacked sufficient context to distinguish normal and abnormal patterns. For these reasons, we adopted the 1 s recording as the basic analysis unit throughout this study.

Through this carefully designed experimental setup, the real-world operational conditions of OLTCs were accurately replicated, providing reliable acoustic data for rigorous evaluation of the performance of the proposed fault diagnosis model.

The proposed model was implemented in PyTorch 2.0 with CUDA 11.7 support and trained on a workstation equipped with a single NVIDIA RTX 3090 GPU (24 GB memory) and an Intel i9-class CPU. The network contained approximately 5.2 million trainable parameters, and the training process required about 2 h for 100 epochs on the OLTC dataset. During inference, the model processed each acoustic scalogram in less than 20 ms, enabling near real-time anomaly detection. This level of computational efficiency highlights the practicality of deploying U-AttentionFlow in online monitoring systems for OLTC fault diagnosis.

**Figure 3:**
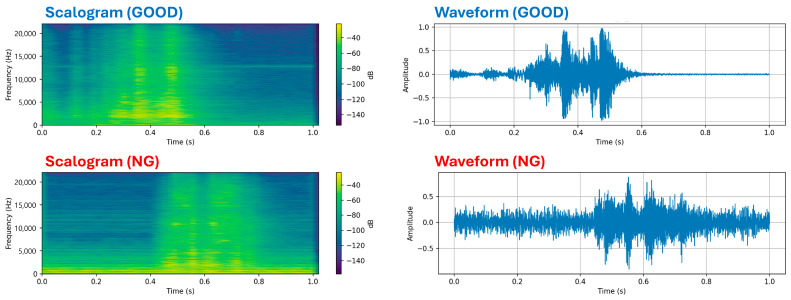
Comparison of Scalograms and Time-Domain Waveforms for GOOD and NG OLTC Switching Signals.

In total, 108,183 normal and 50,362 anomalous data samples were collected. Because the proposed method is a one-class learning network trained exclusively on normal data, only 78,628 normal samples were used for the training set, while the test set comprised 29,555 normal and 50,362 anomalous samples (Table 1, Figure 3). To help the reader visualize the signal characteristics, Figure 3 now presents representative 1 s acoustic recordings from both classes in both the time and time–frequency domains: the top row shows the scalogram and waveform of a properly operating tap-changer, and the bottom row shows those of a defective unit. In the normal case, energy is concentrated in the mid-frequency bands, and the waveform exhibits a smooth, periodic envelope. In the defective case, the waveform has a broader, more irregular envelope and the scalogram displays stronger low-frequency energy with attenuated high-frequency content—features that are indicative of contact wear, bounce, incipient electrical arcing and increased mechanical friction. These frequency-band deviations were therefore adopted as qualitative hallmarks of NG conditions when constructing and interpreting the dataset.

To derive a concrete decision criterion from the observed feature dynamics, we analyzed the distribution of key indicators in the AIR dataset prior to failure. Normal tap-changer operations consistently exhibited spectral centroid values above roughly 1.5 kHz and high-frequency (≥4 kHz) energy ratios above 10%. In contrast, all defective samples showed centroid values well below 1.5 kHz and high-frequency energy ratios that approached zero. We also examined the discrete wavelet transform (DWT) of each 1 s segment. In a six-level DWT decomposition, the signal is split into one approximation band (A6) containing the lowest frequencies and six detail bands (D6–D1) of progressively higher frequency content. Under normal conditions, the DWT scale center—defined as the index of the band contributing the largest proportion of energy—remained within the mid-frequency detail bands (D4–D3), indicating a balance between low- and high-frequency components. As faults developed, energy shifted towards the lower bands: the scale center moved into the lowest two bands and the energy in the A6 band (capturing frequencies below about 340 Hz) rose sharply. Based on these observations, we established the following heuristic thresholds to flag a recording as anomalous: (i) spectral centroid < 1.5 kHz, (ii) high-frequency energy ratio < 10%, and (iii) scale center in one of the two lowest DWT bands with A6 energy exceeding 60%. If a 1 s segment satisfies at least two of these criteria, it is marked as a faulty switch. These empirically derived thresholds translate the qualitative hallmarks of degradation into quantitative rules that can be applied to unseen data.

## 4. Methods

In this study, we aim to achieve robust and accurate anomaly detection using acoustic data from OLTCs. Acoustic data samples were collected at one-second intervals and transformed into two-dimensional image representations through discrete wavelet transforms, generating scalograms. To enhance the anomaly detection and localization performance, the generated 2D scalogram images were processed using a model combining the strengths of the NF and memory-efficient embedding methods. Specifically, we augmented the existing CSFlow architecture, which is known for its superior performance, with additional modules, including SE blocks, a CBAM, MHSA, and a U-Flow-based invertible multi-scale coupling structure.

### 4.1. Audio-to-Image Preprocessing

The continuous wavelet transform (CWT), developed by Morlet and Grossman, represents a time-dependent one-dimensional signal $x(t)\in L^{2}(R)$ as shown in Equation (1).

(1) $$W\left(a,b\right)=\frac{1}{\sqrt{a}}\int_{-\infty }^{\infty }x\left(t\right)\psi^{*}\left(\frac{t-b}{a}\right)dt$$

a is the scale parameter that controls the frequency resolution, and b is the offset parameter that represents time translation. ψ denotes the basis function known as the mother wavelet, and the operator * indicates complex conjugation. Although the CWT offers numerous advantages, most practical signal processing tasks are performed using discrete signals because computer systems inherently handle discrete rather than continuous signals. Therefore, the Discrete Wavelet Transform (DWT) is considered a natural approach, as defined in Equation (2).

(2) $$DWT\left(m,n\right)=\frac{1}{\sqrt{a^{m}}}\sum_{k}s(k)\psi (a^{-m}n-bk)$$

s(k) denotes the input signal, and the DWT provides precise analysis without distorting temporal information, thereby offering significant improvements in signal-processing applications.

A scalogram is a visual representation of the DWT results in the time-frequency domain, illustrating the energy density of the signal. When applying the CWT to a finite discrete time series, discretization of the signal becomes necessary. By choosing an appropriate sampling rate, the CWT can be interpreted as projecting the input signal onto a wavelet ψ, shifted by time t, and scaled by a. The scalogram $S\left(s\right)$ is defined in Equation (3).

(3) $$S\left(s\right)=\left\|Wf(a,b)\right\|={(\int_{-\infty }^{\infty }{\left|Wf(a,b)\right|}^{2}da)}^{\frac{1}{2}}$$

**Figure 4:**
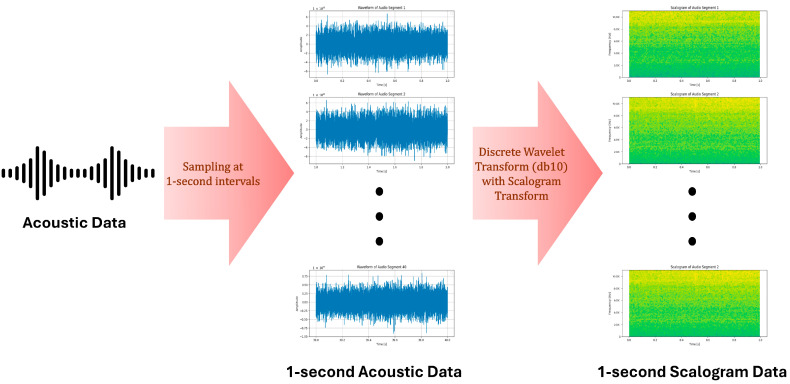
Audio-to-Image Preprocessing.

This equation represents energy at a specific scale, s, and the scalogram allows the detection of the frequencies that contribute most significantly to the total energy of the signal. The complete preprocessing procedure is illustrated in Figure 4.

### 4.2. Base Model: CSFlow

**Figure 5:**
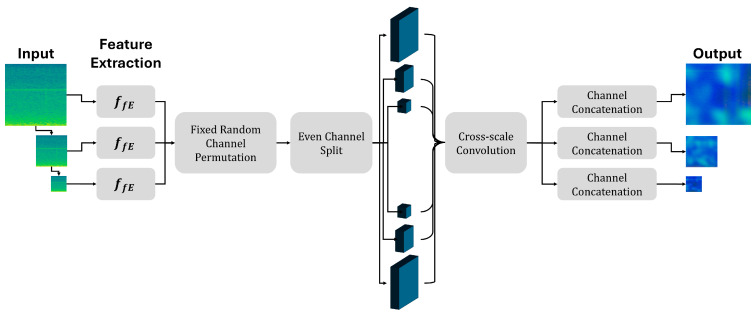
Pipeline for CSFlow.

For image defect detection, CSFlow simultaneous processes feature maps of various scales and exchanges information between these maps to determine the presence of defects Figure 5. In this process, feature maps are transformed according to specific rules, resulting in numerical representations capable of distinguishing between defect-free and defective data. This transformation can be mathematically expressed as shown in Equation (4).

(4) $$f_{csflow}\left(y^{\left(1\right)},\ldots ,y^{\left(s\right)}\right)=\left[z^{\left(1\right)},\ldots ,z^{\left(s\right)}\right]=z\in Z$$

z follows a normal distribution N(0,I). If the transformed numerical value $Pz^{\left(z\right)}$ is less than or equal to a predefined threshold θ, the corresponding image is classified as defective, as expressed in Equation (5).

(5) $$A\left(x\right)=\left\{\begin{array}{l} 1\;if\;Pz^{\left(z\right)}<\theta \\ 0\;otherwise\; \end{array}\right.$$

CSFlow employs coupling blocks based on Real-NVP during the data transformation process. Each block divides the feature map into two parts that mutually influence and transform each other. This transformation process is mathematically described as Equation (6).

(6) $${y_{out,2}=y_{in,2}⊙e^{\gamma_{1}s_{1}\left(y_{in,1}\right)}+\gamma_{1}t_{1}\left(y_{in,1}\right),\;y}_{out,1}=y_{in,1}⊙e^{\gamma_{1}s_{2}\left(y_{out,2}\right)}+\gamma_{2}t_{2}(y_{out,2})$$

Here, ⊙ denotes element-wise multiplication, $s_{1}$, $s_{2}$ adjust the scale of the transformation, and $t_{1}$, $t_{2}$ perform value translation. To ensure numerical stability during the computation, the scale values are constrained, as shown in Equation (7).

(7) $$\sigma_{\alpha }\left(h\right)=\frac{2\alpha }{\pi }arctan\frac{h}{\alpha }$$

The training objective of CSFlow is to accurately model the numerical distribution of the defect-free data. The density of the transformed numerical values z is defined by Equation (8) as follows:

(8) $$p_{Y}\left(y\right)=p_{Z}(z)\left|det\frac{\partial z}{\partial y}\right|$$

Here, $det\frac{\partial z}{\partial y}$ represents the degree of change resulting from the transformation, and is converted into a logarithmic form to be used as the training loss function, as expressed in Equation (9).

(9) $$L\left(y\right)=-\log p_{Y}\left(y\right)=\frac{{\left\|z\right\|}_{2}^{2}}{2}-\log\left|det\frac{\partial z}{\partial y}\right|$$

In addition, CSFlow can visually represent the location of defects within images. The defect scores at each spatial position are computed by aggregating the values across the feature map channels. Higher scores indicate a greater likelihood of defects. Consequently, users can intuitively identify and inspect potential defect regions within the images.

### 4.3. Base Model: U-Flow

**Figure 6:**
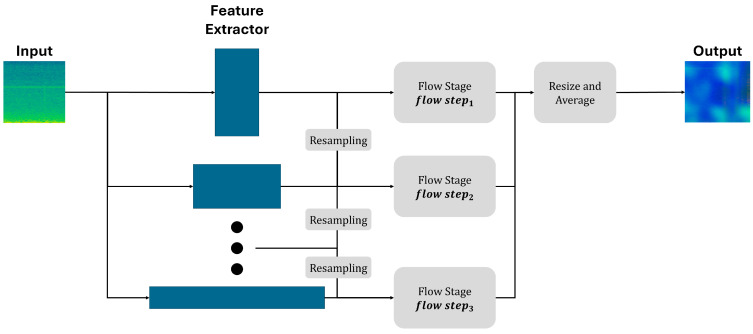
Pipeline for U-Flow.

For anomaly detection in images, U-Flow utilizes a multi-scale Normalizing Flow (NF) architecture designed to effectively model normal data distributions at various resolutions Figure 6. Initially, rich multi-scale image features are extracted through a multi-scale vision Transformer, providing feature representations at different spatial scales. These multi-scale features are then input into a U-shaped invertible NF architecture, similar in structure to a U-Net, enabling hierarchical integration of features across multiple scales.

Within the U-shaped NF framework, each flow stage comprises several affine coupling blocks inspired by RealNVP. Each coupling block splits its input feature map y into two subsets, $y_{1}$ and $y_{2}$, which are transformed interdependently as shown in Equation (10).

(10) $$y_{2,out}=y_{2,in}exp\;⊙\left(s_{1}\left(y_{1,in}\right)\right)+t_{1}\left(y_{1,in}\right),\;y_{1,out}=y_{1,in}exp⊙\left(s_{2}\left(y_{2,out}\right)\right)+t_{2}\left(y_{2,out}\right)$$

where ⊙ denotes element-wise multiplication, and $s_{1}$, $s_{2}$ and $t_{1}$, $t_{2}$ represent scale and translation functions implemented as convolutional neural networks. To maintain numerical stability during training, the scale functions are constrained using Equation (11).

(11) $$\sigma_{\alpha }\left(h\right)=\frac{2\alpha }{\pi }arctan(\frac{h}{\alpha })$$

At each scale, the output of the flow stage is split into two parts; one is directly mapped to a Gaussian latent space, while the other is invertibly upsampled and concatenated with the feature map at the next higher resolution scale. This invertible upsampling operation rearranges pixels and channels to restore higher spatial resolution without loss of information or invertibility.

The resulting latent variables across all spatial positions, channels, and scales are enforced to follow independent standard Gaussian distributions $z\sim Ν\left(o,I\right)$, thereby ensuring statistical independence both within and between scales. Consequently, the joint likelihood across scales factorizes as the product of Gaussian marginal densities. The model training objective maximizes the log-likelihood of normal (defect-free) training data, equivalently minimizing the negative log-likelihood defined as shown in Equation (12).

(12) $$L\left(y\right)=\frac{{\left\|z\right\|}_{2}^{2}}{2}-\log\left|det\frac{\partial z}{\partial y}\right|$$

During inference, anomalies are detected based on low likelihood values in the latent Gaussian space. An unsupervised anomaly detection threshold is automatically derived using an a contrario framework, employing multiple hypothesis testing that leverages the latent independence to control the expected number of false alarms, thus providing reliable and statistically meaningful anomaly segmentation results.

### 4.4. Proposed Model: U-AttentionFlow

**Figure 7:**
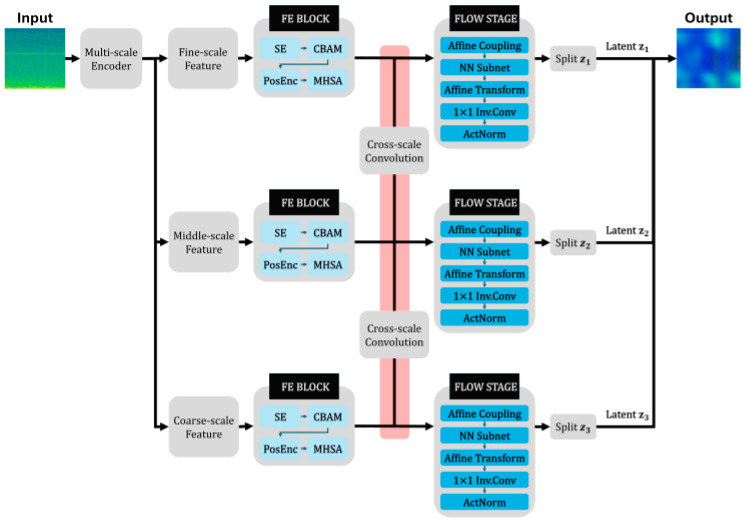
Pipeline for U-AttentionFlow.

In the proposed U-AttentionFlow, the original CS-Flow architecture is enhanced by integrating SE blocks, a CBAM, static positional encoding, MHSA, and a U-Flow-based invertible multiscale coupling module. These enhancements significantly improve the performance of anomaly detection and localization Figure 7.

An SE block was integrated to incorporate channel-wise attention into the CS-Flow architecture. The SE block compresses global spatial information into a compact descriptor and adaptively recalibrates the importance of each feature channel by applying learned scaling coefficients to explicitly “excite” the relevant channels. By leveraging the global context, this mechanism emphasizes informative feature channels while suppressing less relevant channels, thereby guiding the model to focus on defect-sensitive features. The rationale behind employing SE blocks is to enhance subtle anomaly signals that may otherwise be diluted within multiscale feature maps. Through the SE block, channels associated with anomalous patterns are emphasized more clearly and noise is reduced. Consequently, both anomaly detection performance and sensitivity are expected to improve without significantly increasing the model complexity.

For additional feature refinement, a CBAM was integrated into the model. A CBAM is a lightweight attention mechanism that sequentially applies channel and spatial attention to intermediate feature maps. First, the Channel Attention Module (CAM) computes a one-dimensional attention map that signifies channel-wise importance through global pooling and activation functions and subsequently reweighs the feature channels accordingly. Next, the Spatial Attention Module (SAM) aggregates information along the channel dimension and generates a two-dimensional spatial attention map via convolutional operations, highlighting spatially significant areas, and applying this map to feature representations. Through this two-stage refinement process, CBAM explicitly instructs the model regarding where (spatially) and what (channel-wise) to focus on, emphasizing critical regions and channels while suppressing less relevant ones. By integrating the CBAM into the CS-Flow-based model in this study, attention was focused specifically on the features and spatial regions associated with defects. This approach is expected to enhance the detection accuracy of subtle or concealed defects and reduce false activations within normal regions.

To preserve the spatial context, static positional encoding was integrated into the feature-processing stage. Positional encoding assigns a fixed embedding to each spatial location within the feature maps, typically implemented via sine–cosine functions, as proposed by Vaswani et al. [47]. In this study, predetermined sinusoidal positional vectors were added to the features at each spatial location, enabling the model to recognize both absolute and relative positional information explicitly. This is crucial because without positional information, self-attention mechanisms tend to treat features in a permutation-invariant manner, potentially losing the structural information inherent in images. By employing static (nonlearnable) positional encoding, this approach minimizes the additional computational overhead and reduces the risk of overfitting while effectively enhancing spatial awareness. Encoding the positional information enables the flow-based model to distinguish the locations of anomalous patterns more effectively and maintain consistency with the geometric structure of the original image. Consequently, this integration is expected to enable the anomaly detection model to leverage spatial arrangement information, leading to improved accuracy in anomaly identification and localization.

Another significant improvement is the integration of MHSA into the CS-Flow model to effectively capture long-range dependencies. Inspired by transformer networks, MHSA projects feature maps onto multiple sets of queries, keys, and values, and subsequently computes the relationships between spatial positions via self-attention mechanisms. Each attention head learns distinct relationships within the global context, and their outputs are combined in parallel, enabling the modeling of diverse contextual and structural relationships. By incorporating the MHSA into the anomaly detection flow model, the model can dynamically compare the distant regions of the feature maps to determine whether specific areas align with the broader context of the entire image. This is particularly beneficial, as anomalies often cannot be clearly identified based solely on local features but become evident within global contexts such as symmetry or repetitive patterns. Thus, the integration of the MHSA facilitates the learning of global feature interactions, enhancing the accuracy in detecting subtle or context-dependent anomalies, ultimately leading to a more precise anomaly representation and reliable localization of anomalous regions.

Finally, in this study, the conventional NF architecture was redesigned by integrating an invertible multiscale coupling mechanism inspired by the U-Flow structure derived from U-Net. This method enables the combination of multiscale feature information in an invertible manner without information loss. In practical implementations, feature maps are structured at multiple scales, sequentially downsampled into coarse and fine subsets, processed through coupling layers, and subsequently recombined via invertible operations (such as invertible upsampling and channel concatenation). Analogous to U-Net’s skip connections, this approach effectively merges feature information, ensuring no loss of information while maintaining full invertibility. Such a U-Flow-inspired coupling allows for the simultaneous modeling of normal pattern distributions across various resolutions, efficiently leveraging both the global context and detailed local information. The purpose of this design is to accurately capture anomaly patterns of varying scales and effectively integrate multiscale information to precisely detect both structural and subtle local anomalies. Consequently, this structure enables more accurate anomaly detection and localization, facilitating the generation of precise anomaly heat maps that comprehensively utilize integrated multiscale features.

Table 2 summarizes the distinguishing characteristics of U-AttentionFlow, CSFlow and U-Flow. U-AttentionFlow extends the invertible U-shaped flow of U-Flow by inserting squeeze–excitation blocks and the Convolutional Block Attention Module before and after each coupling layer, and it employs static positional encoding and multi-head self-attention to capture temporal context. This configuration allows the model to fuse features across scales through invertible coupling while recalibrating channel and spatial importance and learning long-range dependencies; as a result, it highlights subtle anomalies and preserves multi-scale features, leading to higher accuracy and fewer false alarms. CSFlow uses an invertible flow with cross-scale skip connections but no explicit attention modules; it integrates coarse and fine features through repeated down- and up-sampling, capturing global context and local details, yet its lack of attention makes it less sensitive to fine acoustic anomalies. U-Flow employs an invertible U-shaped architecture without attention; it exchanges information between multiple scales through coupling and skip connections, modelling multi-scale patterns effectively, but the absence of channel and spatial recalibration limits its ability to emphasize subtle defects.

Table 3 outlines the internal structure of U-AttentionFlow in a layer-by-layer fashion. The model takes a 128 × 128 time–frequency scalogram as input. A multi-scale encoder progressively down-samples the input through three convolutional stages to produce fine-, middle-, and coarse-scale feature maps; each convolution uses a 3 × 3 kernel with a stride of 2, halving the spatial resolution and increasing the channel dimension. For each scale, a feature-extraction (FE) block applies three types of attention: a squeeze-and-excitation (SE) block that uses global average pooling followed by two fully connected layers to recalibrate channel importance; a Convolutional Block Attention Module (CBAM) that sequentially performs channel-wise pooling and a 7 × 7 spatial convolution to generate spatial attention; and positional encoding combined with multi-head self-attention (MHSA) to capture long-range temporal dependencies in the scalogram. Cross-scale convolutions then exchange information between adjacent scales while further down-sampling and doubling the number of channels. The flow stage comprises a small neural subnet of two 3 × 3 convolutional layers, an affine transform that shifts and scales feature maps, a 1 × 1 invertible convolution for channel mixing, and ActNorm for per-channel normalization; these operations form an invertible coupling layer that maps the features into latent space. Finally, the latent outputs z_1_, z_2_ and z_3_ are obtained by splitting the features at each scale and are fed into the likelihood computation for anomaly detection. The table shows how each module contributes to extracting, fusing and normalizing multi-scale features, providing a holistic view of the architecture.

In this study, the anomaly detection threshold of U-AttentionFlow was determined using an a contrario framework, following the approach employed in CS-Flow and U-Flow. The model produces log-likelihood values in a latent Gaussian space, where normal samples correspond to higher likelihoods and anomalous samples exhibit lower likelihoods. Based on the distribution of log-likelihood scores obtained from the validation set, the threshold was automatically selected such that the expected number of false alarms (NFA) remained below one. This unsupervised procedure adaptively adjusts the cutoff according to the statistical characteristics of the normal data without the need for manual tuning. Furthermore, considering the practical requirements of OLTC diagnostics, the threshold was configured to eliminate escape cases (missed anomalies), ensuring that abnormal conditions were always detected. All models were evaluated under this zero-escape configuration to provide a fair comparison of performance.

To evaluate and compare the anomaly detection performance using acoustic data, four classification outcomes were considered: True Positive (TP), False Positive (FP), True Negative (TN), and False Negative (FN), obtained by comparing ground-truth labels and model predictions. These can be mathematically represented using Equation (13).

(13) TP=ActualOK∩PredictedOK, FP=ActualNG∩PredictedOK, TN=ActualNG∩PredictedNG, FN=ActualOK∩PredictedNG

In this context, TP corresponds to “OK Detection” in industrial practice, while TN aligns with “NG Detection.” FP is equivalent to “Escape,” and FN corresponds to “Over Kill.” Particularly, in cases of “Escape,” where actual anomalies are incorrectly classified as normal, the system fails to diagnose potentially hazardous equipment conditions. Thus, it is critical to configure deep-learning inspection parameters to minimize the escape rate to zero. Under parameter settings optimized to detect zero-escape cases, we aimed to evaluate the anomaly detection performance by comparing Over Kill outcomes.

## 5. Results and Discussion

In this study, six benchmark models—ReConPatch, PatchCore, PaDiM, MSFlow, FastFlow, CSFlow, and U-Flow—are employed to comparatively evaluate the performance of ReConPatch, PatchCore, and PaDiM are embedding-based approaches that leverage the pretrained CNN features. ReConPatch specializes in fine-grained anomaly detection by learning specialized patch-level embeddings using contrastive learning. PatchCore constructs a memory bank of normal patch embeddings and efficiently identifies anomalies via nearest-neighbor distance calculations. PaDiM spatially models the normal distribution of patches by combining features from multiple CNN layers and utilizes the Mahalanobis distance for anomaly detection. MSFlow, FastFlow, CSFlow, and U-Flow are Normalizing Flow-based methods that transform complex data distributions into clear probability densities to enhance the anomaly detection performance. MSFlow processes multiscale features through parallel flow paths, and effectively detects anomalies of varying sizes. FastFlow rapidly processes single-scale feature maps using a two-dimensional flow, making it well suited for real-time anomaly detection. CSFlow integrates multiscale features into a unified flow model via cross-scale connections, capturing both the global context and local details, and thereby accurately detecting subtle anomalies. U-Flow integrates multiscale features into a unified, fully invertible U-shaped flow architecture via hierarchical connections, effectively modeling statistical independence across scales, and thereby robustly detecting and accurately segmenting subtle anomalies through unsupervised statistical thresholds. These models are widely utilized in diverse industrial applications and exhibit distinct strengths and weaknesses in terms of accuracy, computational speed, and localization performance, based on their respective architectural characteristics.

The performance of the proposed U-AttentionFlow model was compared with those of six state-of-the-art anomaly detection models (ReConPatch, PatchCore, PaDiM, MSFlow, FastFlow, CSFlow, U-Flow), and the results are summarized in Table 4. To assess the robustness of our approach and the baselines, we repeated the random splitting of the dataset into training and testing sets five times, using the same proportions described in Section 3. Each model was retrained and evaluated on each split, and the entries reported in Table 4 correspond to the average performance across these five runs. For a fair comparison, the thresholds for all models were configured to ensure zero Escape instances. The performance evaluation metrics included the correct detection of normal (OK-Detection) and abnormal (NG-Detection) conditions, false alarms (Over-Kill), missed anomalies (Escape), and overall accuracy. Experiments were conducted using an actual transformer OLTC (ECOTAP VPD^®^) under realistic laboratory conditions, employing a three-phase setup with a 12 A load and voltage maintained within a ±5% range of 110 V, replicating genuine OLTC operational scenarios. As indicated in the table, U-AttentionFlow consistently outperformed all the other baseline methods across all metrics. Specifically, U-AttentionFlow achieved the highest accuracy of 99.15%, surpassing existing approaches whose accuracies mostly ranged between 95% and 96%. This superior accuracy clearly demonstrates the capability of the model to correctly classify nearly all the data instances. Moreover, U-AttentionFlow achieved the highest number of correct normal detections (OK-Detection) at 28,873 and simultaneously maintained the lowest number of false alarms (Over-Kill) at only 682. This indicates a substantial reduction in unnecessary alarms due to the misclassification of normal states as abnormal. Furthermore, U-AttentionFlow demonstrated excellent performance in accurately detecting anomalies (NG-Detection), achieving optimal anomaly detection sensitivity, and minimizing missed detections (escape). While all methods exhibited high anomaly detection rates (50,362 cases), the key differentiator was the ability of U-AttentionFlow to effectively eliminate missed anomaly detections. In contrast, conventional methods typically face trade-off issues, either decreasing anomaly detection performance when reducing false alarms or increasing false alarms when enhancing anomaly detection sensitivity. However, U-AttentionFlow simultaneously optimizes sensitivity and accuracy for normal and anomalous conditions, thereby establishing its superior effectiveness and reliability for anomaly detection applications.

The experimental results clearly demonstrate that the proposed U-AttentionFlow model significantly improves the acoustic data-based fault diagnosis performance of OLTCs. Unlike previous approaches, U-AttentionFlow effectively captures complex acoustic patterns by integrating attention mechanisms into a U-Net-inspired normalizing flow architecture capable of multiscale feature integration. Because of this structural advantage, U-AttentionFlow accurately identifies subtle anomalies that traditional methods find difficult to detect, resulting in an exceptionally high accuracy of 99.15%. This performance surpasses the accuracy typically reported in previous studies on acoustic-based fault diagnosis for OLTC, highlighting the excellent generalization capability of our model when applied to real-world operational data. Notably, a major achievement of our approach is the substantial reduction in false alarms (overkill). During testing, U-AttentionFlow produced only 682 false alarms, which was significantly fewer than any of the baseline models compared. Practically, a low overkill rate indicates reduction in unnecessary maintenance or operational disruptions, thereby enhancing system reliability and reducing operational costs. Moreover, the superior performance in correctly classifying normal states (highest OK-detection rate) substantially reduces the likelihood of false alarms during normal operations, increasing operator trust in automated warning systems. U-AttentionFlow also exhibited outstanding capability in accurately detecting actual anomalies (NG-Detection). The minimal rate of missed detections (Escape) is crucial, as it ensures the early identification of critical faults and prevents potentially serious system failures. Some conventional methods exhibit tradeoffs between high anomaly detection rates and high false alarm rates, or vice versa. However, U-AttentionFlow effectively resolves this tradeoff by simultaneously achieving high anomaly detection sensitivity and low false alarm rates, which are critical factors for reliable OLTC condition assessments in operational settings. Given that the experiments were conducted using real OLTC equipment (ECOTAP VPD^®^) under realistic conditions—three-phase 12 A loads and ±5% voltage fluctuations—the results closely reflect actual operational scenarios. Because the performance evaluation was based on high-quality acoustic data collected in controlled laboratory settings, comparable performance is anticipated in field deployments. This indicates a strong potential for the practical application of U-AttentionFlow in OLTC operating environments for predictive maintenance and fault prevention. In summary, the proposed U-AttentionFlow model surpasses existing methods in terms of anomaly detection performance using actual OLTC acoustic data, offering enhanced sensitivity and specificity through its novel approach. Its high-precision diagnostic capability is expected to significantly improve transformer reliability, reduce unnecessary maintenance efforts, and minimize operational downtime, ultimately enhancing overall power grid reliability and efficiency. This empirical demonstration of the advantages conferred by combining multiscale feature flow and attention mechanisms suggests that this model can be beneficially applied to broader power equipment diagnostic applications.

## 6. Conclusions

In this study, we propose a novel deep learning model, U-AttentionFlow, for anomaly detection in OLTCs. The proposed model integrates an attention-based channel and a spatial feature recalibration module consisting of SE blocks and a CBAM. Furthermore, the model incorporates a transformer-based structure that utilizes Static Positional Encoding and MHSA. Additionally, we introduced an invertible multiscale coupling architecture inspired by U-Flow and the U-Net structure, allowing seamless integration of multiresolution feature information without loss. Through these structural innovations, the model effectively captures both local and global patterns, enabling precise detection of subtle anomaly signals. Consequently, U-AttentionFlow significantly enhances the representational power and anomaly detection performance of the model, providing more accurate and reliable diagnoses of OLTC equipment conditions.

The performance of the proposed U-AttentionFlow model was validated through experiments using acoustic data collected directly from an OLTC device (ECOTAP VPD^®^). Under realistic operating conditions, including three-phase 12 A load currents and ±5% voltage fluctuations, the U-AttentionFlow achieved the highest anomaly detection accuracy among all benchmark models. Specifically, the model achieved an accuracy of 99.15%, significantly outperforming existing methods; it typically ranges between 95% and 96%. These experimental results demonstrate the superiority and practical applicability of the proposed approach, confirming stable and reliable anomaly detection, even under actual operational conditions. In other words, U-AttentionFlow simultaneously improves sensitivity and specificity compared with conventional methods, thus providing a highly reliable OLTC anomaly diagnosis capability suitable for real-world deployment.

In future work, we plan to extend the proposed U-AttentionFlow model to an acoustic signal-based predictive diagnosis system to proactively forecast OLTC failures. By leveraging the acoustic signals continuously accumulated during OLTC operations, this approach provides early warnings of potential faults and facilitates preventive maintenance actions. Such a predictive maintenance capability is expected to significantly enhance transformer reliability and improve overall power grid operational safety.

## Figures and Tables

**Table 1:** AIR Dataset Information.

Label	Train	Test
OK	78,628	29,555
NG	0	50,362

**Table 2:** Comparative Overview of U-AttentionFlow, CSFlow and U-Flow.

Model	Architecture	Multi-Scale Feature Processing	Advantages in OLTC Acoustic Anomaly Detection
U-AttentionFlow	Builds on the invertible U-shaped flow of U-Flow and inserts SE blocks and CBAM before/after each coupling layer; employs static positional encoding and multi-head self-attention (MHSA) for temporal context.	Fuses feature across scales using invertible coupling layers; recalibrates channel and spatial importance via SE/CBAM; MHSA captures long-range dependencies and preserves fine details.	The combination of attention modules and invertible multi-scale flow highlights subtle anomalies and preserves multi-scale features, achieving higher accuracy and lower false-alarm rates.
CSFlow	Invertible flow with cross-scale skip connections; does not include explicit attention modules.	Integrates coarse and fine features via cross-scale connections and repeated down/upsampling, maintaining multiscale context.	Cross-scale fusion captures both global context and local details, but the lack of attention makes it less sensitive to fine acoustic anomalies.
U-Flow	Invertible U-shaped architecture; no attention modules.	Exchanges information between multiple scales through invertible coupling and skip connections, preserving features across resolutions.	Effectively models multi-scale patterns but lacks channel/spatial recalibration, limiting its ability to emphasize subtle anomalies.

**Table 3:** Layer Configuration of U-AttentionFlow.

Module/Layer	Input Dimension	Output Dimension	Operation/Kernel	Activation/Notes
Input scalogram	1 × 128 × 128	—	—	1 s DWT scalogram
Multi-scale encoder—fine scale	1 × 128 × 128	C_1_ × 64 × 64	3 × 3 conv, stride 2	ReLU
Multi-scale encoder—middle scale	C_1_ × 64 × 64	C_2_ × 32 × 32	3 × 3 conv, stride 2	ReLU
Multi-scale encoder—coarse scale	C_2_ × 32 × 32	C_3_ × 16 × 16	3 × 3 conv, stride 2	ReLU
FE block—SE	C × H × W	C × H × W	Global average pooling→FC (C→C/16→C)	ReLU, sigmoid; recalibrates channel importance
FE block—CBAM	C × H × W	C × H × W	Channel attention (avg/max pooling), spatial attention (7 × 7 conv)	Sigmoid
FE block—PosEnc + MHSA	C × H × W	C × H × W	Static positional encoding; multi-head self-attention (e.g., 8 heads)	Softmax weighting; captures long-range dependencies
Cross-scale convolution (downsample)	C × H × W	2C × H/2 × W/2	3 × 3 conv, stride 2	ReLU; exchanges information between scales
Flow stage—NN subnet	C × H × W	2C × H × W	Two 3 × 3 conv layers	ReLU
Flow stage—affine transform	2C × H × W	2C × H × W	Element-wise shift and scale	—
Flow stage—1 × 1 invertible conv	2C × H × W	2C × H × W	1 × 1 conv	Channel mixing; invertible
Flow stage—ActNorm	2C × H × W	2C × H × W	Per-channel affine normalization	—
Latent output z_1_ (fine)	—	k_1_	Squeeze and split	Passed to likelihood computation
Latent output z_2_ (middle)	—	k_2_	Squeeze and split	—
Latent output z_3_ (coarse)	—	k_3_	Squeeze and split	—

**Table 4:** Detection Capability Comparison.

Type	Network	Result				
		NG Detection	OK Detection	Overkill	Escape	Accuracy
Memory-efficient Embedding	ReConPatch	25,699	50,362	3856	0	95.17%
	PatchCore	24,849	50,362	4706	0	94.11%
	PaDiM	24,333	50,362	5222	0	93.47%
Normalizing Flow	MSFlow	26,444	50,362	3111	0	96.11%
	FastFlow	26,125	50,362	3430	0	95.71%
	CSFlow	26,754	50,362	2801	0	96.50%
	U-Flow	26,617	50,362	2938	0	96.32%
	U-AttentionFlow	28,873	50,362	682	0	99.15%

## Data Availability

The datasets used and analyzed in the current study are available from the corresponding author upon reasonable request.

## References

[1] Ucar A., Karakose M., Kırımça N. (2024). Artificial Intelligence for Predictive Maintenance Applications: Key Components, Trustworthiness, and Future Trends. Appl. Sci..

[2] Durmus Senyapar H.N., Bayindir R. (2025). The Energy Hunger Paradox of Artificial Intelligence: End of Clean Energy or Magic Wand for Sustainability?. Sustainability.

[3] Butt O.M., Zulqarnain M., Butt T.M. (2021). Recent Advancement in Smart Grid Technology: Future Prospects in the Electrical Power Network. Ain Shams Eng. J..

[4] Panda S., Mohanty S., Rout P.K., Sahu B.K., Parida S.M., Kotb H., Flah A., Tostado-Véliz M., Abdul Samad B., Shouran M. (2022). An Insight into the Integration of Distributed Energy Resources and Energy Storage Systems with Smart Distribution Networks Using Demand-Side Management. Appl. Sci..

[5] Han L., Yin J., Wu L., Sun L., Wei T. (2022). Research on the Novel Flexible On-Load Voltage Regulator Transformer and Voltage Stability Analysis. Energies.

[6] Maseko N.S., Thango B.A., Mabunda N. (2025). Fault Detection in Power Transformers Using Frequency Response Analysis and Machine Learning Models. Appl. Sci..

[7] Tan X., Zhou F., Li W., Ao G., Xu X., Yang L. (2024). Surface Wear Monitoring System of Industrial Transformer Tap-Changer Contacts by Using Voice Signal. Coatings.

[8] Dabaghi-Zarandi F., Behjat V., Gauvin M., Picher P., Ezzaidi H., Fofana I. (2023). Power Transformers OLTC Condition Monitoring Based on Feature Extraction from Vibro-Acoustic Signals: Main Peaks and Euclidean Distance. Sensors.

[9] Ismail F.B., Mazwan M., Al-Faiz H., Marsadek M., Hasini H., Al-Bazi A., Ghazali Y.Z.Y. (2022). An Offline and Online Approach to the OLTC Condition Monitoring: A Review. Energies.

[10] Bustamante S., Lastra J.L.M., Manana M., Alrroyo A. (2024). Distinction between Arcing Faults and Oil Contamination from OLTC Gases. Electronics.

[11] Mariprasath T., Kirubakaran V. (2018). A Real-Time Study on Condition Monitoring of Distribution Transformer Using Thermal Imager. Infrared Phys. Technol..

[12] Gao S., Zhou C., Zhang Z., Geng J., He R., Yin Q., Xing C. (2020). Mechanical Fault Diagnosis of an On-Load Tap Changer by Applying Cuckoo Search Algorithm-Based Fuzzy Weighted LSSVM. Math. Probl. Eng..

[13] Secic A., Krpan M., Kuzle I. (2019). Vibro-Acoustic Methods in the Condition Assessment of Power Transformers: A Survey. IEEE Access.

[14] Dabaghi-Zarandi F., Behjat V., Gauvin M., Picher P., Ezzaidi H., Fofana I. (2024). Using Deep Learning to Detect Anomalies in On-Load Tap Changer Based on Vibro-Acoustic Signal Features. Energies.

[15] Cichoń A., Włodarz M. (2024). OLTC Fault Detection Based on Acoustic Emission and Supported by Machine Learning. Energies.

[16] Han S., Gao F., Wang B., Liu Y., Wang K., Wu D., Zhang C. (2021). Audible Sound Identification of On-Load Tap Changer Based on Mel Spectrum Filtering and CNN. Power Syst. Technol..

[17] Liang X., Wang Y., Gu H. (2022). A Mechanical Fault Diagnosis Model of On-Load Tap Changer Based on Same-Source Heterogeneous Data Fusion. IEEE Trans. Instrum. Meas..

[18] Cichoń A., Borucki S., Włodarz M. (2024). Comparative Analysis of Acoustic Emission Signals from On-Load Tap-Changers for Potential Detecting of Non-Simultaneous Operations. Arch. Acoust..

[19] Saveliev A., Tomanović D., Fuhr J., Stanisavljević M. (2024). Detection of On-Load Tap-Changer Contact Wear Using Vibroacoustic Measurements. IEEE Trans. Power Deliv..

[20] Ezzaidi H., Fofana I., Picher P., Gauvin M. (2024). On the Feasibility of Detecting Faults and Irregularities in On-Load Tap Changers by Vibroacoustic Signal Analysis. Sensors.

[21] Secić A., Aizpurua J.I., Garro U., Muxika E., Kuzle I. (2025). Transformer OLTC Operation Monitoring Framework through Acoustic Signal Processing and Convolutional Neural Networks. IEEE Trans. Instrum. Meas..

[22] Ruff L., Vandermeulen R., Goernitz N., Deecke L., Siddiqui S.A., Binder A., Müller E., Kloft M. Deep One-Class Classification. Proceedings of the 35th International Conference on Machine Learning (ICML).

[23] Gong D., Liu L., Le V., Saha B., Mansour M.R., Venkatesh S., Hengel A.V.D. Memorizing Normality to Detect Anomaly: Memory-Augmented Deep Autoencoder for Unsupervised Anomaly Detection. Proceedings of the IEEE/CVF International Conference on Computer Vision (ICCV).

[24] Park H., Noh J., Ham B. Learning Memory-Guided Normality for Anomaly Detection. Proceedings of the IEEE/CVF Conference on Computer Vision and Pattern Recognition (CVPR).

[25] Kirichenko P., Izmailov P., Wilson A.G. (2020). Why Normalizing Flows Fail to Detect Out-of-Distribution Data. Advances in Neural Information Processing Systems (NeurIPS).

[26] Papamakarios G., Nalisnick E., Rezende D.J., Mohamed S., Lakshminarayanan B. (2021). Normalizing Flows for Probabilistic Modeling and Inference. J. Mach. Learn. Res..

[27] Ataeiasad F., Elizondo D., Calderón Ramírez S., Greenfield S., Deka L. (2024). Out-of-Distribution Detection with Memory-Augmented Variational Autoencoder. Mathematics.

[28] Yamada T., Ikeura R., Suzuki T., Shibata T., Takemura K. Efficient Anomaly Detection via Matrix Sketching-Based Memory-Efficient Embeddings. Proceedings of the AAAI Conference on Artificial Intelligence.

[29] Huang J., Gong Y., Yuan H. (2023). Memory-Efficient Deep Autoencoder with Sparse Embeddings for Unsupervised Anomaly Detection. IEEE Trans. Neural Netw. Learn. Syst..

[30] Zhong T., Qin C., Shi G., Xue Y., Ma Y. (2024). A residual denoising and multiscale attention-based weighted domain adaptation network for tunnel boring machine main bearing fault diagnosis. Sci. China Technol. Sci..

[31] Jonschkowski R., Stone A., Barron J.T., Gordon A., Konolige K., Angelova A. What Matters in Unsupervised Optical Flow. Proceedings of the European Conference on Computer Vision (ECCV).

[32] Dosovitskiy A., Beyer L., Kolesnikov A., Weissenborn D., Zhai X., Unterthiner T., Dehghani M., Minderer M., Heigold G., Gelly S. An Image is Worth 16 × 16 Words: Transformers for Image Recognition at Scale. Proceedings of the International Conference on Learning Representations (ICLR).

[33] Liu Z., Lin Y., Cao Y., Hu H., Weti Y., Zhang Z., Lin S., Guo B. Swin Transformer: Hierarchical Vision Transformer Using Shifted Windows. Proceedings of the IEEE/CVF International Conference on Computer Vision (ICCV).

[34] Mishra P., Verk R., Fornasier D., Piciarelli C., Foresti G.L. VT-ADL: A Vision Transformer Network for Image Anomaly Detection and Localization. Proceedings of the IEEE International Symposium on Industrial Electronics (ISIE).

[35] Lee Y., Kang P. (2022). AnoViT: Unsupervised Anomaly Detection and Localization with Vision Transformer-Based Encoder-Decoder. IEEE Access.

[36] Tailanian M., Pardo Á., Musé P. (2024). U-Flow: A U-Shaped Normalizing Flow for Anomaly Detection with Unsupervised Threshold. J. Math. Imaging Vis..

[37] Hu J., Shen L., Sun G. Squeeze-and-Excitation Networks. Proceedings of the IEEE Conference on Computer Vision and Pattern Recognition (CVPR), Salt Lake City.

[38] Woo S., Park J., Lee J.-Y., Kweon I.S. CBAM: Convolutional Block Attention Module. Proceedings of the European Conference on Computer Vision (ECCV).

[39] Hyun J., Kim S., Jeton G., Kim S.H., Bale K., Kang B.J. ReConPatch: Contrastive Patch Representation Learning for Industrial Anomaly Detection. Proceedings of the IEEE/CVF Winter Conference on Applications of Computer Vision (WACV).

[40] Roth K., Pemula L., Zepeda J., Scholkopf B., Brox T., Gehler P. Towards Total Recall in Industrial Anomaly Detection. Proceedings of the IEEE/CVF Conference on Computer Vision and Pattern Recognition (CVPR).

[41] Defard T., Setkov A., Loesch A., Audigier R. PaDiM: A Patch Distribution Modeling Framework for Anomaly Detection and Localization. Proceedings of the International Conference on Pattern Recognition (ICPR).

[42] Rudolph M., Wandt B., Rosenhahn B. Same Same but Differnet: Semi-Supervised Defect Detection with Normalizing Flows. Proceedings of the IEEE/CVF Winter Conference on Applications of Computer Vision (WACV).

[43] Gudovskiy D., Ishizaka S., Kozuka K. CFLOW-AD: Real-Time Unsupervised Anomaly Detection with Localization via Conditional Normalizing Flows. Proceedings of the IEEE/CVF Winter Conference on Applications of Computer Vision (WACV).

[44] Yu J., Zheng Y., Wang X., Li W., Wu Y., Zhao R., Wu L. (2021). FastFlow: Unsupervised Anomaly Detection and Localization via 2D Normalizing Flows. arXiv.

[45] Zhou Y., Xu X., Song J., Shen F., Shen H.T. (2023). MSFlow: Multi-Scale Flow-Based Framework for Unsupervised Anomaly Detection. arXiv.

[46] Rudolph M., Wehrbein T., Rosenhahn B., Wandt B. Fully Convolutional Cross-Scale-Flows for Image-Based Defect Detection. Proceedings of the IEEE/CVF Winter Conference on Applications of Computer Vision (WACV).

[47] Vaswani A., Shazeer N., Parmar N., Uszkoreit J., Jones L., Gomez A.N., Kaiser Ł., Polosukhin I. Attention Is All You Need. Proceedings of the 31st Conference on Neural Information Processing Systems (NIPS 2017).

